# Targeting Myc Interacting Proteins as a Winding Path in Cancer Therapy

**DOI:** 10.3389/fphar.2021.748852

**Published:** 2021-09-29

**Authors:** Yihui Zhou, Xiaomeng Gao, Meng Yuan, Bo Yang, Qiaojun He, Ji Cao

**Affiliations:** ^1^ Zhejiang Province Key Laboratory of Anti-Cancer Drug Research, Institute of Pharmacology and Toxicology, College of Pharmaceutical Sciences, Zhejiang University, Hangzhou, China; ^2^ The Innovation Institute for Artificial Intelligence in Medicine, Zhejiang University, Hangzhou, China; ^3^ Cancer Center of Zhejiang University, Hangzhou, China

**Keywords:** MYC, cancer therapy, interaction protein, transcriptional regulation, post-translational modification, inhibitors

## Abstract

*MYC*, as a well-known oncogene, plays essential roles in promoting tumor occurrence, development, invasion and metastasis in many kinds of solid tumors and hematologic neoplasms. In tumors, the low expression and the short half-life of Myc are reversed, cause tumorigenesis. And proteins that directly interact with different Myc domains have exerted a significant impact in the process of Myc-driven carcinogenesis. Apart from affecting the transcription of Myc target genes, Myc interaction proteins also regulate the stability of Myc through acetylation, methylation, phosphorylation and other post-translational modifications, as well as competitive combination with Myc. In this review, we summarize a series of Myc interacting proteins and recent advances in the related inhibitors, hoping that can provide new opportunities for Myc-driven cancer treatment.

## 1 Introduction

Myc is a multifunctional transcription factor, regulates multiple genes comprised of varieties of cell physiological and pathological processes including proliferation, differentiation, apoptosis and tumorigenesis (Farrell and Sears, 2014). Originally *MYC* was isolated on chicken cells, and the gene encoding c-Myc was a cellular homolog of v-Myc, which was present in avian myelocytomatosis virus strain 29 causing avian leukemia (Vennstrom et al., 1982). Subsequently other transformed and more specific Myc family members were also identified in mammal tissues, including c-Myc, N-Myc and L-Myc, respectively (Dalla-Favera et al., 1982; Nau et al., 1985; Rickman et al., 2018). Myc family genes have been shown to be differentially expressed in terms of tissue type and developmental stage (Xu et al., 1991). c-Myc only express in tissues with rapid proliferation, while L-Myc and N-Myc often express specifically in tissues that undergoing differentiation (Hirning et al., 1991). Besides, the mice lack of c-Myc or N-Myc all lead to embryonic death (Pirity et al., 2006). In comparison, L-Myc is only unnecessary for gross morphological development, by *MYCL* knockout mice model. This might be due to the overlapping expression patterns of other Myc has made up for L-Myc deficiency (Hatton et al., 1996).

Although there are three types of Myc and their chromosomal locations are different, they are all homologous proteins, which are highly conserved in gene sequence and have similar structural domains (Chen et al., 2018). Myc has several structure regions that are critical for the biological functions, including the amino-terminal transactivation domain (TAD), central region and the carboxy-terminal basic-helix-loop-helix-leucine zipper (bHLH-LZ) domain (Duffy et al., 2021). bHLH-LZ domain is responsible for dimerization with its essential partner, Myc-associated protein X (Max), and for sequence-particular DNA binding. TAD and central region are main protein-protein interaction (PPI) area, including six highly conserved regions (MB0, MBI, MBII, MBIIIa, MBIIIb, MBIV), termed Myc homology boxes (MBs). MB0 accelerates the transcription by binding to the general transcription factor IIF (TFIIF); MBI controls proteasome-mediated degradation of Myc protein; MBII participates in chromatin remodeling and modification; MBIIIa play a role in gene repression; MBIIIb binds to WD repeat domain 5 (WDR5) as a glue binding on chromatin and MBIV shows potential association with chromatin (including apoptosis, G2 cell arrest) (Baluapuri et al., 2020; Duffy et al., 2021). Through proteomics analysis, more than half of the Myc interactors demand at least one of MBs for binding (Kalkat et al., 2018).

It is now clear that Myc proteins are principal drivers of human tumorigenesis, more than 70% of cancers are related to Myc disorders (Dang et al., 2006; Lancho and Herranz, 2018). Minor alteration of Myc levels can facilitate or prevent oncogenic transformation and tumour progression (Wang T. et al., 2019). Myc binds to the promoters of downstream genes at the RNA polymerase II (RNAPII)-bound and promotes their expression, regulating the increase or decrease of transcription. The carcinogenicity of Myc is that, it can increase the transcription level of high-affinity target genes or even push them to saturation, and can also regulate (up-regulate or down-regulate) low-affinity target genes, transforming normal cells into tumor cells (Baluapuri et al., 2020). *MYC* gene is activated mainly through amplification and chromosomal translocation rearrangement. It can regulate the expression of a variety of genes related to cell proliferation and metabolic process, and its corresponding genes are also the most common high abundance oncogenes in human cancers (Difilippantonio et al., 2002; Chen et al., 2014). Myc protein is expressed at a low level in proliferating cells and has a very short half-life of only 30 min, after which it is degraded by the ubiquitin proteasome pathway (Thomas and Tansey, 2011). However, this characteristic of Myc is often changed in tumors, prolonged half-life and excessive accumulation are also a major cause of promoting the occurrence of tumors (Wu et al., 2020).

The process of Myc binding to the target chromatin and regulating the transcription level of the target gene is not completed independently. The well-known protein Max, which is first described as Myc-interacting protein (Blackwood and Eisenman, 1991). Max binds to the bHLH-LZ domain of Myc and forms Myc/Max heterodimers to achieve DNA recognition and binding (Cascón and Robledo, 2012). In most chromatin binding and transcriptional regulation, Myc is entirely dependent on heterodimerization with Max (Grandori and Eisenman, 1997; Castell et al., 2018). Deletion of Max destabilizes Myc protein and reduces the expression of Myc-target gene, even eliminates Myc-driven tumorigenesis (Mathsyaraja et al., 2019; Augert et al., 2020). Recent evidence showed, Myc still retained some biological functions without Max, meaning Max was not the only interacting protein that maintains Myc functions (Cascón and Robledo, 2012). Besides of Max, some other critical proteins can interact with Myc as well to regulate physiological processes including transcription activation, transcription repression, chromatin remodeling and ubiquitination degradation, etc.

**FIGURE 1:**
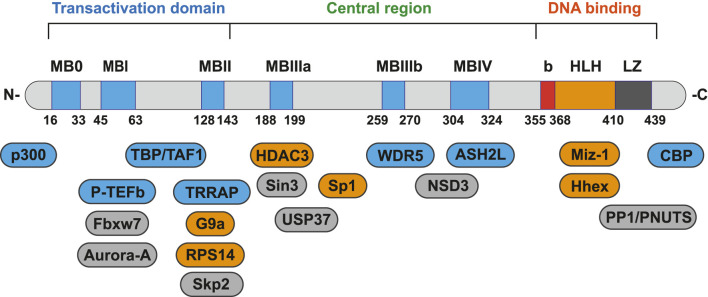
Protein-protein interaction on Myc domains.

This review, we concentrate on a number of Myc interacting proteins that contribute to Myc function (Figure 1), and also discussed current inhibitors and strategies targeting the interacting proteins, in the interest of providing new opportunities for Myc-related cancer treatment.

## 2 Protein-Protein Interaction Works on Myc Transcriptional Activation

**FIGURE 2:**
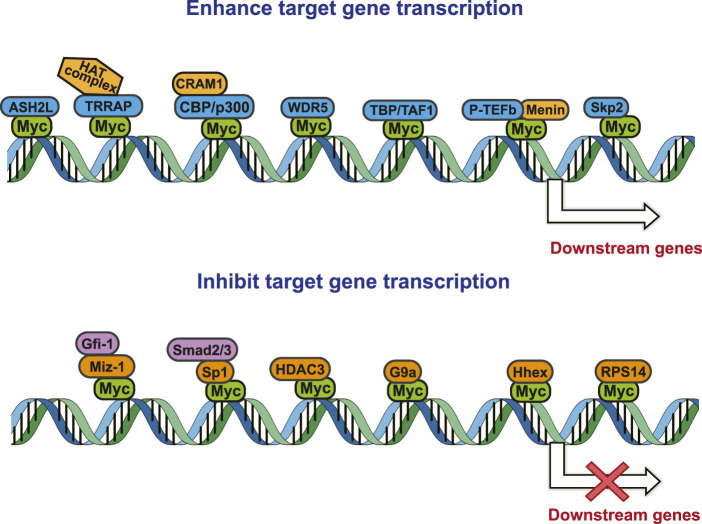
Protein-protein interaction works on Myc transcriptional activation and repression.

As a transcriptional regulator, Myc affects a wide range of gene transcription levels. Under normal circumstances, the excessive growth and proliferation of Myc-amplified tumors are caused by the transcriptional activation of oncogenes by Myc (Kim et al., 2019). The bHLH-LZ DNA binding domain of Myc binds to chromatin and recruits some cofactor proteins to modify the chromatin or Myc itself, and finally achieve the function of chromatin transcription activation (Tu et al., 2015). In this process, the interacting proteins play a decisive role in coordination with the function of Myc (Figure 2).

### 2.1 Transactivation/Transformation-Domain Associated Protein (TRRAP)

TRRAP is a component of histone-acetylation (HAT) complexes, acts as a scaffold to stabilize (Cogné et al., 2019). Although being part of the phosphoinositide 3-kinase-related kinase (PIKK) family, TRRAP lacks a kinase domain (Elías-Villalobos et al., 2019). It was reported that TRRAP has direct interaction with Myc in the MBⅡ domain, and the recruitment of TRRAP was required for Myc-mediated oncogenic transformation (Nikiforov et al., 2002). In HAT complexes, Tat-interactive protein 60 (Tip60) and General control non-derepressible 5 (Gcn5) work histone acetylase activity, and TRRAP itself doesn’t exert catalytic activity (Liu et al., 2003; Feris et al., 2019). TRRAP links between HAT complexes and Myc, enables the activities of HAT complexes to be recruited and anchored at Myc binding DNA areas in order to stimulate gene expression (McMahon et al., 2000). After recruitment by TRRAP, HAT complexes regulate the modification of histones near promoter and hyperacetylation of lysine residues on terminal of histones, creating an open chromatin environment to promote transcription (Kalkat et al., 2018). Without serum stimulation, for low level of H4 acetylation, Myc alone was inefficient in inducing target genes’ expression (Frank et al., 2001). In addition, reducing the acetylase activity of Tip60 affects the function of the HAT complex, and the level of Myc binding to chromatin will also be weakened (Frank et al., 2003). Therefore, as a cofactor of myc, TRRAP can not only promote the binding of Myc to chromatin, but also open up the nearby chromatin environment to promote transcription.

### 2.2 cAMP-Response-Element-Binding Protein (CBP/p300)

Acetyltransferases p300 and CBP are multifunctional transcriptional co-activators, belonging to lysine acetyltransferases (KATs) family. Due to their extensive sequence homology and functional similarity, they are defined as a whole: CBP/p300 (Weinert et al., 2018). CBP/p300 contains a catalytic domain KAT to acetylate target proteins, and a recognition domain bromodomain (BRD) to bind with the acetylated proteins. For this reason, CBP/p300 can not only be recruited by MYC to modify chromatin acetylation, but also regulate the acetylation level of Myc itself. Six lysine residues in Myc are direct substrates of p300, and acetylated Myc could interact with promoter binding factors as Miz-1 effectively (Zhang et al., 2005). A recent study reported that p300 binds to c-Myc N-terminus and recruit co-activator-associated arginine methyltransferase 1 (CARM1), in which CARM1-p300-c-Myc-Max (CPCM) transcriptional complex controls the transcription of *CUL4A/4B* (Lu et al., 2020). Interestingly, CBP binds to the carboxy-terminal region of c-Myc without transactivating activity. This modification is no need MBII, indicating that this function is independent of TRRAP (Vervoorts et al., 2003).

### 2.3 WD Repeat Domain 5 (WDR5)

With highly conserved WD40 repeat-containing protein, WDR5 is indispensable for appropriate regulation of multi-cellular processes (Guarnaccia and Tansey, 2018). WDR5 protein comprises seven WD40 repeat domains, folding into a seven-bladed propeller structure with several exposed surfaces (Lu et al., 2018). WDR5 mainly exists in the histone lysine methyltransferase subclass 2 (KMT2) enzymes and the non-specific lethal (NSL) complex (Guarnaccia et al., 2021). On account of unusual structure and exposed surfaces, WDR5 forms multiprotein complexes, including with Myc. Acting as a cofactor, WDR5 contributes to the recruitment of Myc to chromatin, and directly combines with Myc on its shallow hydrophobic cleft (Thomas et al., 2019). Myc interacts with WDR5 *via* an evolutionarily conserved MBIIIb domain, and the core amino acid sequence is “-EEIDVV-” (Thomas et al., 2015b). Otherwise, WDR5 controls Myc target gene transcription by inducing demethylation and subsequently acetylation of H3K27 (Ullius et al., 2014). Myc-WDR5 interaction stabilizes Myc/Max dimer on the promotor of pivotal protumorigenic target genes, accelerating the process of gene transcription (Thomas et al., 2015a). WDR5 could interact with the MBIIIb motif of c-Myc and facilitate Myc-induced *HIF1-*α transcription, therefore promoting the EMT, invasion and metastasis of cholangiocarcinoma (CCA) (Chen et al., 2021). Myc also maintains the DNA replication in pancreatic ductal adenocarcinoma (PDAC) cells appropriately through interacting with WDR5 (Carugo et al., 2016).

### 2.4 TATA-Binding Protein (TBP)

TBP is an essential component of the transcription initiation complex TFIID, participating in most gene expression processes in eukaryotes (Bhuiyan and Timmers, 2019). TBP and Myc have been reported to interact at two sites, both of which are located in the TAD domain of Myc. TBP combines at 115–124 amino acids of Myc, and TBP-associated factor 1 (TAF1) at 98–111 (Wei et al., 2019). Studies have shown that the Myc-TBP interaction enhanced gene transcription by regulating the energy distribution upon the transcription initiation complex assembly (Wei et al., 2019). TBP stimulates the transcriptional activation of Myc and enhances the functional characteristics of Myc target genes (Barrett et al., 2005).

### 2.5 Positive Transcription Elongation Factor b (P-TEFb)

P-TEFb is a transcription factor that stimulates transcription elongation by RNAPII, and functions through directly interacting with various cellular transcription factors, leading to a variety of inflammatory diseases and tumors (Fujinaga, 2020). P-TEFb is composed of the cyclin-dependent kinase 9 (Cdk9) and its regulatory subunit cyclin T. Cdk9 in P-TEFb can phosphorylate the C-terminal domain (CTD) of RNAPII (Kanazawa et al., 2003). While Cyclin T1 binds to Myc at the highly conserved region MBI, promoting the function of Myc to activate the cad promoter (Eberhardy and Farnham, 2002). Menin interacts with TAD domain of Myc and cyclin T1, and subsequently enhances Myc-mediated transcription *via* P-TEFb (Wu et al., 2017). The cooperation between P-TEFb and Myc also requires the Ski-interacting protein (SKIP), an mRNA elongation and splicing factor (Brès et al., 2009).

### 2.6 Set1/Ash2 Histone Methyltransferase Complex Subunit ASH2 (ASH2L)

ASH2L is a transcriptional regulator, as part of the KMT2 complex it is involved in methylation and dimethylation at “Lys-4” of histone H3. Research showed, ASH2L and Myc directly interacted *in vitro* and existed chromatin co-location. Two distinct domains in Myc play to ASH2L binding, 263–350 amino acids directly and bHLH-LZ domain indirectly (Ullius et al., 2014). Since both ASH2L and WDR5 are subunits of KMT2 complex, Myc does not recruit ASH2L to participate in chromatin binding, so the interaction between Myc and ASH2L may be guided by WDR5. Knockdown of ASH2L affects transcription of Myc target genes (Ullius et al., 2014).

## 3 Protein-Protein Interaction Works on Myc Transcriptional Repression

Tumor occurrence is often accompanied by mutations and abnormal expressions of proto-oncogenes as well as tumor suppressor genes. Upon regulating target genes and promoting cancer progression, Myc not only promotes the transcription of oncogenes, but also suppresses the transcription of tumor suppressor genes. During the tumor-promoting process, the MBII domain and bHLH-LZ domain are necessary for Myc to inhibit transcription, and there are numerous interacting proteins helpful to exert this function. Besides, there are interacting proteins binding to other Myc domains, which can also affect this process (Figure 2).

### 3.1 Myc Interacting Zinc Finger Protein 1 (Miz-1)

Miz-1, a transcription factor containing BTB/POZ domain, can come into play as an activator or repressor depending on its binding partners (Möröy et al., 2011). Recent research suggested that the transcriptional activities of c-Myc can be reversed once associated with Miz-1. Miz-1 competes with Max to form a complex with c-Myc through the b-HLH-LZ domain (between 12th and 13th zinc finger) (Bédard et al., 2017). Miz-1 can interact with zinc-finger (ZF) transcriptional repressor growth factor independence 1 (Gfi-1) and Myc, form a ternary complex at the cyclin dependent kinase inhibitor (CDKN) promoter (including CDKN1A and CDKN2B), and repress CDKN synergistically (Basu et al., 2009; Liu et al., 2010; Aesoy et al., 2014). Myc is directly recruited by Miz-1 to the cell cycle inhibitors p15^INK4B^ and p21^CIP1^ promoter, inhibits tumour suppressor p53 and favours the initiation of apoptosis (Seoane et al., 2002; Qi et al., 2017). The Mad family is known as an endogenous transcription suppressor of Myc due to its interaction with Max, Mad4 also is suppressed by Miz1-Myc complex (Quéva et al., 1998). In addition, c-Myc contributes to Wnt inhibitory factor-1 (WIF-1) transcriptional repression in a Miz-1-dependent manner (Licchesi et al., 2010). In leukemia stem cells (LSCs), Myc-Miz-1 interaction represses the expression of CCAAT/enhancer-binding protein α (Cebpα) and Cebpδ, accelerating the self-renewal of LSCs (Zhang et al., 2020). Ablation of the Miz-1 POZ domain conduces to treatment of leukemias and lymphomas, chemotherapy more effective with targeting Miz-1 (Ross et al., 2019).

### 3.2 Specificity Protein 1 (Sp1)

Sp1 is a significant transcription factor, through specific binding to GC-rich DNA sequences, regulates the expression of polytype genes (Vizcaíno et al., 2015). For promoting the transcription of tumor-related growth factors, Sp1 expressed high level in kinds of tumors and associated with poor prognosis (Beishline and Azizkhan-Clifford, 2015). By interacting with Myc on central region (143–352), Sp1-Myc can repress p21 transcription, thus covering the p21-mediated cell cycle checkpoint (Gartel et al., 2001). Myc can also bind to the Sp1/Myc overlapping site, inhibits the promoter activity and endogenous mRNA expression of *BRD7* (Liu et al., 2008). Through the Sp1-Smad complex at the promoter of *CDKN2B*, Smad2 and Smad3 can directly interact with Myc. Thus affect the transcriptional activity of Sp1 and Sp1-Smad-dependent transcription of the *CDKN2B* (Feng et al., 2002; 2016).

### 3.3 Histone Deacetylase 3 (HDAC3)

As a member of the Class I HDAC family, HDAC3 assists the acetyl groups removed on histone and non-histone, repressing gene transcription by promoting chromatin contraction (Dávalos-Salas et al., 2019). HDAC3 interacts with Myc through the MBIIIa domain (Kurland and Tansey, 2008), and subsequently reduces miR-15a/16-1 level in mantle cell lymphoma (MCL) by anchoring at the two promoters of the miR-15a/16-1 cluster gene, *DLEU2*, and exerting repressive function (Zhang et al., 2012). Tumor necrosis factor receptor-associated factor 6 (TRAF6) can ubiquitinate HDAC3 and lead to the dissociation of HDAC3 from the c-Myc, and then promote human hepatocarcinogenesis (Wu et al., 2020). HDAC3-Myc induces *FOXA2* transcriptional repression through its regulation on FOXA2-mediated FTO/m6A/MYC axis, leading to the development of gastric cancer (Yang et al., 2021).

### 3.4 G9a

G9a is a primary enzyme that catalyzes the methylation of histone 3 lysine 9 (H3K9) and histone 3 lysine 27 (H3K27), playing a crucial role in diverse biological processes and human diseases (Chen et al., 2017; Cao et al., 2019). The MBII region has been identified essential for Myc-G9a interaction, which could promote breast tumor growth by inhibiting gene transcription. Without G9a, H3K9me2 level decreased at Myc-repressed gene promoters, and reduced Myc binding loci (Tu et al., 2018). Meanwhile, depletion of G9a *in vivo* suppresses Myc-dependent tumor growth. Deficiency of G9a reduces c-Myc binding activity to promoters and inhibits glioblastoma cell proliferation and tumorigenesis ability (Ke et al., 2020). Dual EZH2 and G9a inhibition suppresses multiple myeloma (MM) cell proliferation through the IRF4-Myc axis (Ishiguro et al., 2021). It is worth mentioning that Myc-G9a repress gene transcription in Miz-1-independent manner, this reminds that G9a is necessary for Myc chromatin-binding and gene repression (Tu et al., 2018).

### 3.5 Haematopoietically Expressed Homeobox (Hhex)

Hhex is a transcriptional repression regulator mainly in charge of organismal development and hematopoiesis (Goh et al., 2020). Hhex can regulate the proliferation level of NK cells and cooperate with the corepressor transducin-like enhancer of Split3 (Tle3) to promote memory B cells (MBCs) development (Laidlaw et al., 2020). In addition to the positive regulation of normal cells, Hhex also negatively regulate the differentiation and function of Treg cells via inhibition of *Foxp3* (Jang et al., 2019). Recent research has shown that Hhex was able to interact with the bHLH-LZ region of c-Myc. Hhex overexpression limits the transcription activation, hyperproliferation, metabolism activity and transformation characteristic of Myc oncogenic activities by disrupting Myc/Max formation (Marfil et al., 2015). It is foreseeable that Hhex could be used as a new negative regulator of Myc to inhibit its carcinogenic ability.

### 3.6 Ribosomal Protein S14 (RPS14)

The demonstration of haploinsufficiency of RPS14 is recognized one of the reasons for p53 activation, and RSP14 is also associated with cellular senescence (Rhoads and Roufa, 1991; Boultwood, 2011). Recent research found that RPS14 affected the transcription function of Myc. RPS14 interacts with MBII and the bHLH-LZ domains of the oncoprotein c-Myc, and prevents the recruitment of Myc-cofactor TRRAP (Zhou et al., 2013). RPS14 not only directly inhibits c-Myc transcriptional activity, but also reduces c-Myc mRNA level (Zhou et al., 2013).

## 4 Protein-Protein Interaction Works on Myc Protein Stability

**FIGURE 3:**
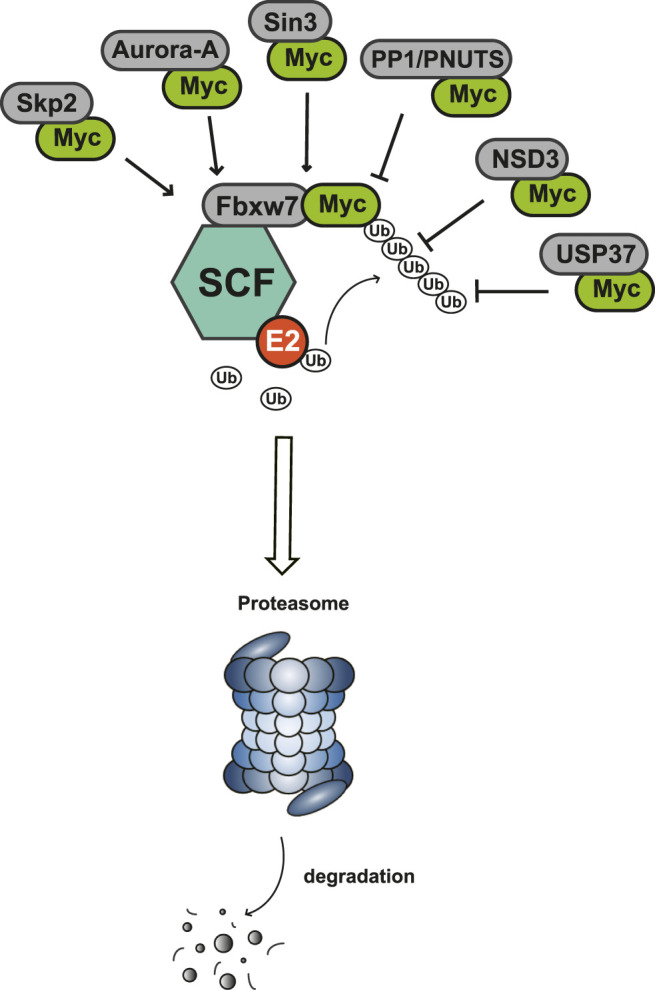
Protein-protein interaction works on Myc degradation.

Myc is unstable in cells, with short half-life of ∼30 min (Cattoretti, 2013; De Melo et al., 2017). The degradation of Myc is mainly dependent on the phosphorylation of serine-62 and threonine-58 in MBI region by cyclin B/Cdk1 and Gsk3 sequentially, and both of these two residues are often mutated in cancer (Yada et al., 2004). The phosphorylation of Ser62 and Thr58 touches off protein phosphatase 2A (PP2A)-mediated Ser62 dephosphorylation (Mudgapalli et al., 2019). In normal cells, the most important way to control Myc levels is through the targeted degradation of the ubiquitin-proteasome system (UPS). UPS consists of ubiquitin (Ub), ubiquitin activase (E1), ubiquitin-conjugating enzyme (E2), ubiquitin ligase (E3), proteasome and its substrate (Asmamaw et al., 2020). The substrate K48 site was ubiquitinated by E1, E2 and E3, and the ubiquitinated protein was degraded by proteasomes. In *MYC*-driven cancers, due to the mutation or overexpression of Myc, the proteasome is not enough to degrade Myc any more, leading to excessive accumulation of Myc and eventual tumorigenesis (Bahram et al., 2000). According to existing research, some interacting proteins have been reported to affect the phosphorylation modification of Myc protein, and subsequently affect the degradation of Myc through Fbxw7-mediated ubiquitination modification (Figure 3).

### 4.1 F-Box With 7 Tandem WD40 (Fbxw7)

The Fbxw7 encoded by *FBXW7* is one of the crucial components of Skp1-Cullin1-F-box (SCF) complex, which targets proteins for UPS degradation (Sailo et al., 2019). Fbxw7 interacting and subsequently destabilizing with Myc relies on the phosphorylation of MBI: modifying Myc with K48-linked ubiquitin chains, leading to poly-ubiquitylation and the degradation of Myc through UPS (Welcker et al., 2004; Yada et al., 2004). In embryonic stem cell, Fbxw7 controls its differentiation by degrading c-Myc (Buckley et al., 2012). Loss of Fbxw7 cooperating with activated Akt to induce c-Myc-dependent cholangiocarcinogenesis in mice (Wang J. et al., 2019). In T cell acute lymphoblastic leukemia, Fbxw7 mutations affect the half-life of c-Myc and strengthen leukemia initiating cell activity (King et al., 2013). In addition, deubiquitinating enzyme (DUB) USP9X antagonizes Fbxw7 ubiquitylation to regulate Fbw7 protein stability, reduces c-Myc and alleviates tumor progression (Khan et al., 2018). And DUB USP28 stabilizes c-Myc may also via Fbxw7 complex (Gersch et al., 2019).

### 4.2 S-phase Kinase-Associated Protein 2 (Skp2)

Skp2 was discovered as a partner of the CDK2 complex at first, but identified as the F-box-binding component of the SCF complex later. Skp2 triggers c-Myc ubiquitylation through directly interacting with the MBII (Hydbring et al., 2017). The interaction of Skp2-Myc occurs at stages from G1 to S phase in normal lymphocytes (von der Lehr et al., 2003). Interestingly, Skp2 is a transcriptional co-activator for Myc as well, considered to be an essential component for recognizing Myc activation domain and activating Myc target genes (Kim et al., 2003). Therefore, Skp2 has positive effect in the interaction with Myc from two aspects, which is achieved by combining with different Myc domains.

### 4.3 Aurora-A

Aurora-A is a serine/threonine kinase of the Aurora kinase family, including Aurora-A, Aurora-B, and Aurora-C (Yan et al., 2016). Aurora-A is a powerful oncogene that has been reported to promote tumor proliferation, invasion and metastasis through mitosis and other ways (Li et al., 2018; Lin et al., 2020). Aurora-A interacts with N-Myc on both sides of MBI, upon which the ubiquitin ligase Fbxw7 complexes also bind with N-Myc (Richards et al., 2016). Aurora-A-N-Myc protects N-Myc from proteasomal degradation mediated by the Fbxw7, thus inhibits N-Myc degradation and stabilizes the protein level of N-Myc (Otto et al., 2009). On the other hand, high level of Aurora-A enhances the expression and transcriptional activity of c-Myc, and c-Myc can regulate the transcription level of Aurora-A in turn (den Hollander et al., 2010; Yang et al., 2010). Therefore, Aurora-A is likely to be an important Myc stability regulator, which can also affect the transcriptional activation ability of Myc.

### 4.4 Protein Phosphatase 1 (PP1)/Protein Phosphatase-1 Nuclear-Targeting Subunit (PNUTS)

PP1 is a Ser/Thr phosphatase, and PNUTS is a regulatory subunit of PP1 (Wang F. et al., 2019). PP1 catalyzes the dephosphorylation of more than half of phosphorylated serine and threonine in cells (Bertolotti, 2018). The binding area of PP1/PNUTS with Myc is still uncertain, but it can be observed that the enrichment of Myc-Max and Myc-PP1/PNUTS on Myc target gene promoters (Dingar et al., 2018). By proximity ligation assay (PLA), endogenic Myc-PNUTS interaction was defineded (Dingar et al., 2018). Inhibition of PP1/PNUTS induced the hyperphosphorylation of Myc, causing degradation by the classical SCF-Fbxw7 pathway (Dingar et al., 2018). In addition, PNUTS knockdown resulted in decreased N-Myc protein, and repressed the progression of *MYCN*-amplified neuroblastoma (Tee et al., 2020). So PP1/PNUTS is also asignificant assistant of Myc’s carcinogenic process.

### 4.5 Sin3

Sin3 is a transcriptional repressor with a similar structure of the helix-loop-helix dimerization domain from Myc (Kadamb et al., 2013). Sin3 forms a complex with HDAC, thus regulates histone deacetylation and gene transcription (Banks et al., 2020). Sin3 includes Sin3a and Sin3b, both of which can interact with Myc (Yang et al., 2012; Garcia-Sanz et al., 2014). Sin3b interacts with Myc at amino acids 186–203, belonging to the MBIIIa domain, and recruits HDAC1 to exert the deacetylase activity (Garcia-Sanz et al., 2014). However, Sin3 itself is not associated with Myc target gene down-regulation, only inducing the degradation of Myc, while the transcriptional repression of Myc needs to combine with Mad-Max or Mxi1-Max complexes (Harper et al., 1996).

### 4.6 Nuclear Receptor Binding SET Domain Protein 3 (NSD3)

NSD3 is a histone lysine methyltransferase, identified as a Myc cofactor (Li et al., 2017). A noncatalytic isoform of NSD3, named NSD3S, shows specially stabilization of Myc half-life. NSD3S binds directly with Myc domain between MBIII and MBIV, and NSD3S residues 389–404 plays a functional role in it. NSD3S suppresses the FBXW7 activity by interacting with Myc, increases Myc half-life and transcriptional function (Gonzalez-Pecchi et al., 2020).

### 4.7 Ubiquitin-Specific Protease 37 (USP37)

USPs that may regulate c-Myc stability, like USP9X and USP28, stabilizes c-Myc *via* Fbxw7 (Popov et al., 2007; Khan et al., 2018). USP37 as a novel deubiquitinating enzyme (DUB) that binds c-Myc directly to stabilize it. USP37 binds with Myc MBIII domain, stabilizes c-Myc from polyubiquitination-mediated degradation independent of Fbxw7 (Pan et al., 2015). In lung cancers, USP37 expression is upregulated and positively correlated with Myc, suggests that USP37-Myc inhibitors may be a therapeutic strategy for lung cancer.

## 5 Inhibitor Progression of Protein-Protein Interaction With Myc

Myc inhibitors designed based on protein-protein interactions have been studied. In addition, in the process of research on other proteins inhibitors that existed directly Myc-interaction, it has also been found to have an impact on the function of Myc and the stability of the protein. These inhibitors may be a new weapon against the oncogene *MYC* (Table 1).

**TABLE 1:** Inhibitors and functions of Myc interaction proteins.

Interaction protein	Inhibitor	Function on Myc	Structural formula	References
CBP/p300	CPI-637	Binds to bromodomain of CBP/p300, inhibits *MYC* expression	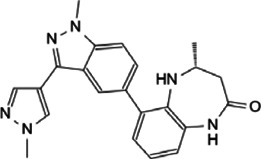	Taylor et al. (2016)
	NEO2734	Induces depletion of Myc and inhibition of multiple myeloma growth	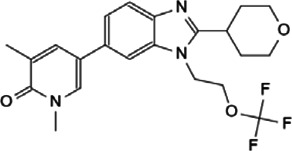	Spriano et al. (2020)
	NEO1132		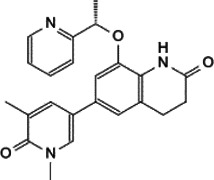	
WDR5	Compound 12	Strongly interrupts the interaction of WDR5-Myc complex	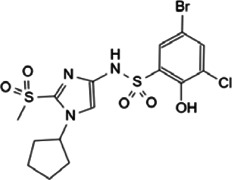	Chacón Simon et al. (2020)
	Compound 16	Reduces Myc recruitment to chromatin at WDR5-Myc co-bound genes	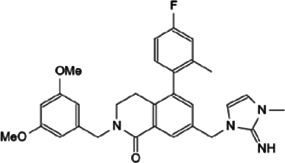	Tian et al. (2020)
P-TEFb	KL-1	Downregulates Myc and transcriptional regulated by Myc by destroying the P-TEFb complex	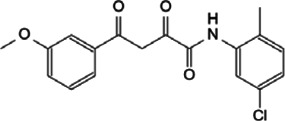	Liang et al. (2018)
	KL-2		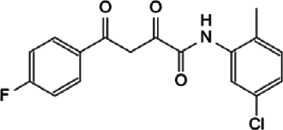	
	CYC065	Hinders the transcriptional activation of N-Myc by inhibiting the Cdk9 of P-TEFb	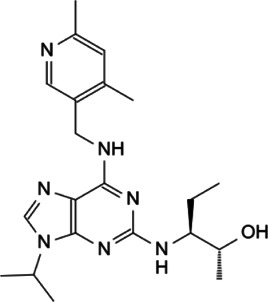	Poon et al. (2020)
	Atuveciclib (BAY 1143572)	Inhibits phosphorylation of RNAPII and reduces Myc level	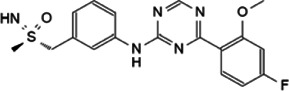	Lücking et al. (2017); Narita et al. (2017)
	UNC10112785	Destabilizes and induces the substantial loss of Myc protein	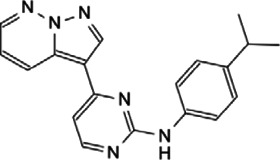	Blake et al. (2019)
	SNS-032	Represses the c-Myc-dependent transcription of *RhoA* gene	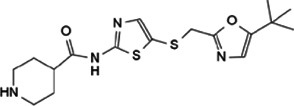	Zhang et al. (2019)
HDAC	Vorinostat (SAHA)	Induces c-Myc acetylation at lysine 323, disrupts Myc’s transcriptional repression	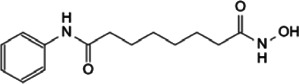	Nebbioso et al. (2017)
	entinostat (MS27-275)		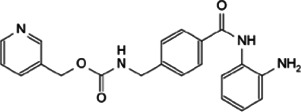	
	Panobinostat (LBH589)	Reduces Myc protein level	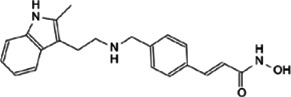	Beyer et al. (2019)
	RGFP966	Remits Myc-mediated transcriptional repression of the miR-15 and let-7 families in malignant cells	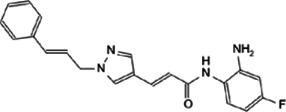	Konstantinopoulos et al. (2006); Adams et al. (2016)
	depsipeptide (FK228)		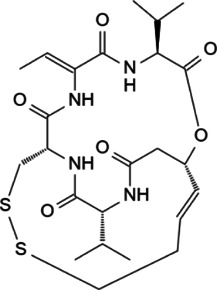	
	CD532	Breaks the native conformation of Aurora-A and drives the degradation of N-Myc protein	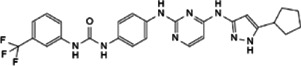	Gustafson et al. (2014)
Aurora-A	Alisertib	Disrupts the N-Myc-Aurora-A complex, inhibits N-Myc signaling	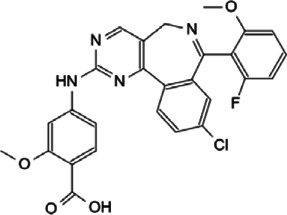	Beltran et al. (2019)
	CCT137690	Reduces N-Myc protein in a dose-dependent manner	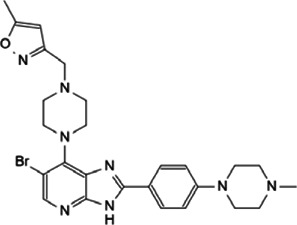	Ommer et al. (2020)

### 5.1 Targeting TRRAP-Myc Interaction

As *TRRAP* is an essential gene, mutation or deletion of *TRRAP* leads to early embryonic lethality or poor embryonic development (Iwanami et al., 2009; Shukla et al., 2011). Due to the importance of TRRAP in the organism, knocking out or degrading TRRAP is not a good way to treat Myc-amplified tumors (Leduc et al., 2014). Blocking or interrupting the PPIs between TRRAP and Myc can inhibit the transcriptional activation of Myc. What’s more, MBII is interaction interface of TRRAP and Myc, both form of a structurally-stable conformation, thus the development of Myc-PPIs inhibitors targeting the MBII domain is an effective strategy (Feris et al., 2019). Besides, ribosomal proteins L11 shows inhibition on c-Myc -induced transcription and cell proliferation by competing with TRRAP upon binding to MBII (Dai et al., 2007a; Dai et al., 2007b). Silencing of L11 increased the expression level of Myc (Jung et al., 2016). Therefore, TRRAP-Myc inhibitors can be designed based on the L11 protein structure.

### 5.2 Targeting CBP/p300-Myc Interaction

Targeting lysine acetyltransferases CBP/p300 is an effective strategy, small molecule inhibitors that target some of these PPIs domains have been developed. Aiming at the bromodomain of CBP/p300, inhibition probe CPI-637 strongly inhibits *MYC* expression (Taylor et al., 2016). Inhibitors like NEO2734 and NEO1132 targeting both BET and CBP/p300 proteins could induce the depletion of Myc and inhibition of multiple myeloma growth (Spriano et al., 2020). Sensitivity to the dual inhibitors was only in connection with Myc protein expression levels (Ryan et al., 2021).

### 5.3 Targeting WDR5-Myc Interaction

Not only c-Myc, all Myc family members could interact with WDR5. In *MYCN*-amplified neuroblastomas, WDR5 functions as a core cofactor participating in transcriptional activation and tumorigenesis under the guidance of N-Myc. Clinically, high expression of WDR5 in neuroblastoma were a valid indicator of unfavorable prognosis (Sun et al., 2015). It is suggested that the strategy of inhibiting Myc through WDR5 can be adopted to treat a variety of malignant tumors (Thomas et al., 2015b). WDR5 has two main active pockets, a hydrophobic cleft: WDR5 binding motif (WBM) and an arginine-binding pocket: WDR5 interaction (WIN) site (Macdonald et al., 2019; Bryan et al., 2020). A preponderant small molecule inhibitor of the WDR5-Myc interaction based on WDR5 WBM-site structure is compound 12 (Chacón Simon et al., 2020). This compound disrupted the WDR5-Myc interaction in cell lysates, and co-IP in HEK293 cells showed a ∼4-fold reduction of the WDR5-Myc with treating compound 12. Besides, a novel WDR5 WIN site antagonist containing a dihydroisoquinolinone bicyclic core is designed, named compound 16 (Tian et al., 2020). Compound 16 reduces Myc recruitment to chromatin and inhibits Myc–driven cancer proliferation.

### 5.4 Targeting P-TEFb-Myc Interaction

The development of Cdk9 inhibitors is an advantageous strategy for the P-TEFb-Myc interaction. Up to now, multiple Cdk9 inhibitors have been developed, some of which can affect the transcription function of Myc, weaken the stability of Myc and promote Myc degradation. Peptidomimetic lead compounds, KL-1 and KL-2, downregulates Myc and transcriptional regulated by Myc by destroying the P-TEFb complex (Liang et al., 2018). A clinical inhibitor of Cdk9 and Cdk2, CYC065, can hinder the transcriptional activation of N-Myc by inhibiting the Cdk9 in P-TEFb complex, realizing the therapeutic effect on MYCN-amplified neuroblastoma (Poon et al., 2020). Atuveciclib (BAY 1143572) is a highly selective P-TEFb/Cdk9 inhibitor, which inhibits the phosphorylation of RNAPII and reduces Myc level (Lücking et al., 2017; Narita et al., 2017). UNC10112785 is a potent Cdk9 inhibitor, that destabilizes Myc and induces the substantial loss of Myc protein in KRAS-mutant pancreatic cancer (Blake et al., 2019). A Cdk7/9 inhibitor SNS-032 represses the c-Myc-dependent transcription of *RhoA* gene, inhibiting liver metastasis in uveal melanoma (Zhang et al., 2019). However, long-term inhibition of Cdk9 may also lead to a compensatory increase in Myc expression and recruit more P-TEFb to Myc target genes in the end (Lu et al., 2015). This suggests that we need to use combination therapy for long-term treatment of tumors when targeting Cdk9.

### 5.5 Targeting HDAC-Myc Interaction

Histone deacetylase inhibitors (HDACi) is a kind of anti-tumor drug with great development potential. HDAC is that can target Myc mainly selectively inhibit HDAC1 and HDAC3. The HDACi Vorinostat (SAHA) and Entinostat (MS27-275) are effective against leukemic cells, which could induce c-Myc acetylation at lysine 323 and disrupt Myc’s transcriptional repression, finally inducing *TRAIL* expression and apoptosis (Nebbioso et al., 2017). Panobinostat (LBH589) is a pan-HDACi, which could reduce Myc protein level in human AML cell lines (Beyer et al., 2019). The HDAC3 inhibitor RGFP966 and HDAC1/2 inhibitor depsipeptide (FK228) remit Myc-mediated transcriptional repression of the miR-15 and let-7 families in malignant cells, inducing apoptosis as a result (Konstantinopoulos et al., 2006; Adams et al., 2016).

### 5.6 Targeting Aurora-A-Myc Interaction

For the reason that the presence of Aurora-A increases the stability of Myc, inhibitors targeting Aurora-A can promote the degradation of Myc and achieve the effect of tumor inhibition. An Aurora-A inhibitor CD532 breaks the native conformation of Aurora-A and drives the degradation of N-Myc in N-Myc-driven cancers (Gustafson et al., 2014). The stronger evidence is that CD532 can cause cells blocking entry into S-phase and lead a subsequent G0/G1 arrest, which is a phenomenon of damaged Myc function (Gustafson et al., 2014). Alisertib is an oral Aurora kinase inhibitor, that has entered clinical trials for a variety of diseases (DuBois et al., 2018; O'Connor et al., 2019; Gay et al., 2020). Alisertib consistently disrupted the N-Myc-Aurora-A complex *in vitro*, thus inhibited N-Myc signaling and suppressed tumor growth (Beltran et al., 2019). CCT137690 is a potent inhibitor of Aurora kinases, which could dose-dependent reduce N-Myc protein level in Rhabdomyosarcoma (RMS) cells (Ommer et al., 2020).

## 6 Discussion

There are ample evidences that manifests targeting Myc could form the element of extensively effective anti-cancer therapies. However, due to the flat structure of Myc, there is no binding pocket for moleculars, making the idea of directly inhibiting Myc difficult to become a reality. Some researchers have tried to exploit small molecule drugs to break the Myc/Max interaction, but the feasibility is limited. One difficulty is that there is extensive contact of the bHLH-LZ domain. And a large number of transcription factors share this motif. Therefore, it is arduous to separately inhibit Myc/Max heterodimer without causing off-target side effects on other transcription factors bound to bHLH-LZ. Eventually produce great toxic side effects on normal cells.

What’s more, it turns out that Myc’s recognition of target genes not only depend on the interaction with Max. Model shows that in terms of the affinity of Myc/Max dimers to DNA, about 90% of Myc binding cases in cells cannot be interpreted, and it has been shown that many nucleoproteins can promote Myc recruitment to its target genes (Lorenzin et al., 2016). Some of these recruited proteins interact directly with Myc, and the other proteins form a protein complex which participate in the regulation of Myc function and protein stability. More and more studies have shown that there are many transcription cofactors, which either affect or even determine the transcription of target genes by Myc through protein post-translational modification, or change protein conformation, or compete for protein binding sites. If inhibitors can be designed based on such protein-protein interactions, targeting Myc interacting proteins can achieve the goal of curing Myc-amplified tumors. From the information we summarized in this review, we can see that a variety of small molecule and peptide inhibitors have shown more or less effect on the protein expression level, degradation level, and target gene transcription level of Myc. Some inhibitors were designed from the beginning to destroy the protein interaction of Myc protein. They have indeed achieved certain results in preclinical or clinical trials, which can effectively inhibit tumor growth and promote tumor apoptosis.

Of course, these inhibitors also face some problems. First, for the reason that the targeted proteins are in charge of multiple physiological functions in cells, the inhibition of these proteins may also have an impact on other cell functions. Second, whether the inhibitor can accurately target the Myc-interacting protein complex and how selective it is, remain to be verified. Third, due to the powerful ability of Myc itself, although the capacity of a single inhibitor was strong, will there be any compensation or replacement, making the final therapeutic effect insignificant? These problems have yet to be resolved.

On the whole, the most important point is that targeting the direct PPIs between Myc and other cofactor proteins is an effective and feasible strategy for the treatment of diseases caused by Myc in spite of existing thorny problems mentioned above. We believe that targeting Myc interacting proteins could become a winding path in Myc-associated cancer therapy in the future.
