# Corrigendum: Targeting Myc Interacting Proteins as a Winding Path in Cancer Therapy

**DOI:** 10.3389/fphar.2021.785732

**Published:** 2021-10-20

**Authors:** Yihui Zhou, Xiaomeng Gao, Meng Yuan, Bo Yang, Qiaojun He, Ji Cao

**Affiliations:** ^1^ Zhejiang Province Key Laboratory of Anti-Cancer Drug Research, Institute of Pharmacology and Toxicology, College of Pharmaceutical Sciences, Zhejiang University, Hangzhou, China; ^2^ The Innovation Institute for Artificial Intelligence in Medicine, Zhejiang University, Hangzhou, China; ^3^ Cancer Center of Zhejiang University, Hangzhou, China

**Keywords:** MYC, cancer therapy, interaction protein, transcriptional regulation, post-translational modification, inhibitors

In the published article, there was an error regarding the **Affiliation** for **Meng Yuan**. Instead of “Cancer Center of Zhejiang University, Hangzhou, China,” the correct affiliation is “The Innovation Institute for Artificial Intelligence in Medicine, Zhejiang University, Hangzhou, China.”

The authors apologize for this error and state that this does not change the scientific conclusions of the article in any way. The original article has been updated.

